# Protists with Uncertain Phylogenetic Affiliations for Resolving the Deep Tree of Eukaryotes

**DOI:** 10.3390/microorganisms13081926

**Published:** 2025-08-18

**Authors:** Euki Yazaki, Takashi Shiratori, Yuji Inagaki

**Affiliations:** 1 Research Center for Advanced Analysis, National Agriculture and Food Research Organization, Tsukuba 305-0856, Ibaraki, Japan; 2 Institute of Life and Environmental Sciences, University of Tsukuba, Tsukuba 305-8572, Ibaraki, Japan; shiratori.takashi.gm@u.tsukuba.ac.jp; 3 Center for Computational Sciences, University of Tsukuba, Tsukuba 305-8577, Ibaraki, Japan; yuji@ccs.tsukuba.ac.jp

**Keywords:** protists with uncertain phylogenetic affiliations, PUPA, eukaryotic tree of life, phylogenomics

## Abstract

Resolving the eukaryotic tree of life (eToL) remains a fundamental challenge in biology. Much of eukaryotic phylogenetic diversity is occupied by unicellular microbial eukaryotes (i.e., protists). Among these, the phylogenetic positions of a significant number of lineages remain unresolved due to limited data and ambiguous traits. To address this issue, we introduce the term “PUPAs” (protists with uncertain phylogenetic affiliations) to collectively describe these lineages, instead of using vague or inconsistent labels, such as *incertae sedis* or orphan taxa. Historically, protists were classified based solely on morphological features, and many with divergent cell structures were left unplaced in the eToL. With the advent of sequence-based approaches, the phylogenetic affiliations of some PUPAs have been clarified using molecular markers, such as small subunit ribosomal DNA. The combination of technological progress and continuous efforts to cultivate diverse protists, including PUPAs and novel protists, now enables phylogenetic analyses based on hundreds of proteins, providing their concrete placements in the eToL. For example, these advances have led to the discovery of new deep-branching lineages (e.g., Hemimastigophora), the resolution of relationships among major groups (e.g., *Microheliella*, which linked Cryptista and Archaeplastida), and insights into evolutionary innovations within specific clades (e.g., *Glissandra*). In this review, we summarize current consensus in eukaryotic phylogeny and highlight recent findings on PUPAs whose phylogenetic affiliations have been clarified. We also discuss a few lineages for which the phylogenetic homes remain unsettled, the evolutionary implications of these discoveries, and the remaining challenges in resolving the complete eToL.

## 1. The Potential Role of Protists with Uncertain Phylogenetic Affiliations in the Elucidation of the Eukaryotic Tree of Life

The history of biology spans more than 2300 years, beginning with the systematic study of living organisms in Aristotle’s *History of Animals* and *Parts of Animals*, where he conducted detailed empirical observations and sought to generalize biological principles [1,2]. In recent decades, technological innovations—particularly in high-resolution microscopy, high-throughput sequencing, and computational methods, including artificial intelligence—have dramatically accelerated the pace of discovery, opening new frontiers in our understanding of life’s complexity. Yet, despite the long history of biological inquiry and remarkable advances in methodology, many fundamental questions in biology remain unresolved. For example, in the study of cellular structures and dynamics, the mechanisms underlying the formation of membrane-less compartments via liquid–liquid phase separation are still not fully understood [3]. Similarly, in intracellular trafficking, critical aspects of pathway selection and regulation, such as those involved in autophagy, continue to be actively investigated [4]. In developmental biology, although the advent of induced pluripotent stem (iPS) cell technology has revolutionized the field, the molecular switches that govern early cell fate decisions remain elusive, as do the relationships between chromatin state and transcriptional regulation [5]. Among these enduring questions, understanding biological diversity, particularly the diversity of eukaryotes, remains a major scientific challenge. The aspiration to reconstruct the evolutionary history of all life on Earth into a single, unified tree of life dates back to Ernst Haeckel, who, inspired by Darwin’s theory of evolution, made the first attempt to visualize such a framework [6]. Today, it is widely accepted that all extant organisms belong to one of three domains—Bacteria, Archaea, and Eukarya [7], although recent phylogenomic studies suggest that Eukarya emerged from within Archaea, effectively supporting a two-domain model of life [8,9,10,11]. The latest analyses using 30 conserved protein markers have shown a specific affiliation between eukaryotes and the Heimdallarchaeota–Wukongarchaeota lineage [11]. However, the relationships among these domains and the internal phylogenetic structures within each domain remain subjects of ongoing revision and debate [8,12,13,14,15,16]. Among these, the internal phylogeny of eukaryotes is especially complex, and our understanding of the eukaryotic tree of life (eToL) remains incomplete.

Resolving the eToL requires performing robust molecular phylogenetic analyses with appropriate datasets sampled from broad eukaryotic lineages. In this context, uncovering the full extent of eukaryotic diversity—by identifying and characterizing representatives from across the major lineages—is not only essential but effectively synonymous with resolving the eToL itself. On a global scale, the vast majority of eukaryotic diversity is represented by unicellular microbial eukaryotes, collectively known as protists. In contrast to multicellular eukaryotes, such as animals (Metazoa) and plants (Archaeplastida), which are confined to a limited number of clades, protists span a broad range of evolutionary lineages. Therefore, protists are central to any comprehensive effort to resolve the eToL [17,18]. Despite an estimated global biomass (~4 Gt) that exceeds that of animals (~2 Gt) [19], the majority of protists remain poorly characterized due to their microscopic size and the technical challenges associated with their cultivation.

**Figure 1:**
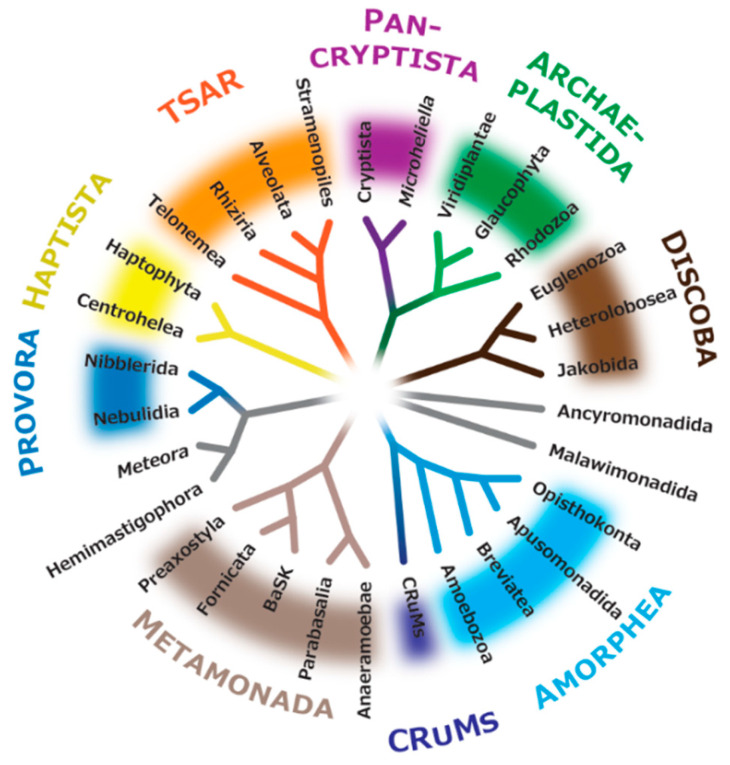
**Schematic overview of the eukaryotic tree of life.** Based on the results from the phylogenomic analyses published as of July 2025, eukaryotes are coalesced into nine major supergroups or supergroup-compatible clades.

Sustained efforts by protist taxonomists, however, have gradually advanced our understanding of protist diversity. Since the 1990s, the widespread use of molecular phylogenetic analyses, particularly those based on small subunit ribosomal RNA genes (SSU rDNA), has led to substantial revisions of protist taxonomy and a more nuanced understanding of overall eukaryotic diversity [20,21,22,23]. More recently, single-cell sequencing and multi-locus phylogenetic analyses (phylogenomic analyses) have further accelerated the resolution of deep phylogenetic relationships among eukaryotes. These advances have successive revisions of higher-level eukaryotic classification systems, such as those published by the International Society of Protistologists in 2005, 2012, and 2019 [24,25,26]. We present the schematic eToL updated by the results from the latest phylogenomic studies in Figure 1. Despite these achievements, the deep evolutionary relationships among eukaryotic lineages remain contentious [15,27,28]. A key obstacle is the existence of numerous protists with uncertain phylogenetic affiliations. Traditionally, these organisms have been referred to as *incertae sedis* or “orphans.” However, the former term is unfamiliar outside of communities studying taxonomy, and the latter term has lacked clear definitions and is not easily interpretable based on the particular wording alone. In this review, we propose to call it a “protist with uncertain phylogenetic affiliation” or a “PUPA,” which defines and refers to such an organism more explicitly than *incertae sedis* or an orphan. The term is an acronym derived from the initial letters of the phrase, and its plural form is set to “PUPAs.” This heterogeneous category includes organisms whose placement cannot be confidently resolved by conventional markers, such as SSU rDNA, and morphologically described taxa that lack molecular data altogether. Many PUPAs are difficult to culture or maintain under laboratory conditions, often resulting in the loss of cells before thorough characterization can be completed.

Here, we provide a comprehensive overview of recent advances in studying PUPAs and assess their significance for resolving the eToL. PUPAs may represent previously unrecognized branches of the eToL and are likely crucial for resolving early divergences in eukaryotic evolution [28,29,30,31,32,33,34,35]. Systematically elucidating the phylogenetic positions of PUPAs can bridge gaps in our current understanding of eukaryotic diversity and evolution. In sum, we regard elucidating the phylogenetic positions of PUPAs as indispensable for constructing a comprehensive and accurate eToL. To our knowledge, no previous review has consolidated recent key findings across the protists, including PUPAs, that updated the diversity and evolution of eukaryotes. This review aims to unify the recent progress in resolving the eToL, which may help elucidate the pivotal innovations in cellular and genome evolution in eukaryotes and guide future research priorities related to PUPAs and novel protists, including targeted sampling and sequencing efforts.

## 2. Changes in Protist Classification and PUPAs

**Figure 2:**
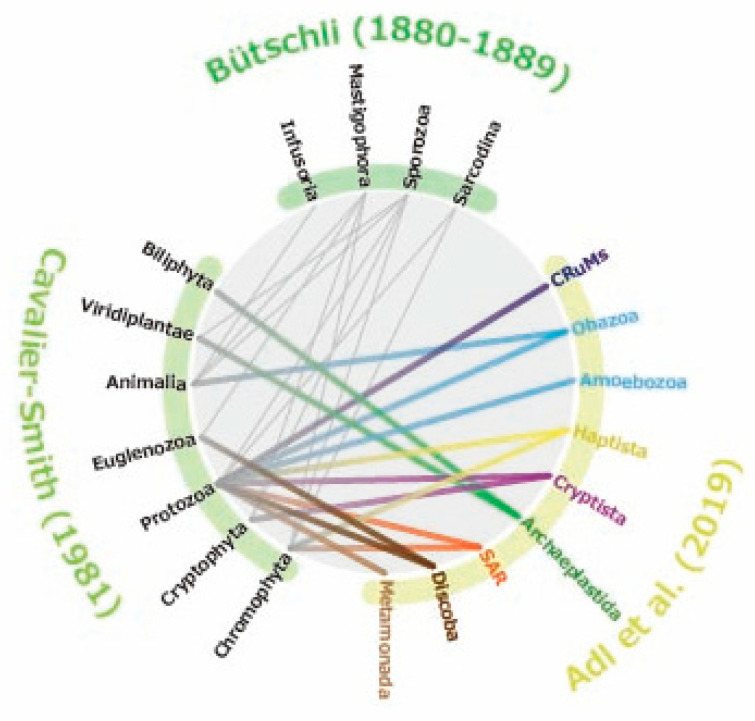
**Development in classification systems of eukaryotes.** Lines indicate correspondence between taxonomic groups across different classification systems [26,45].

Since Antonie van Leeuwenhoek first observed microorganisms under the microscope in the 17th century, protists have been described and classified primarily based on observations made with light microscopes. For centuries, these organisms were incorporated into the traditional dichotomous classification system of the Animal and Plant kingdoms. In early microscopical studies by Müller (1786) [36] and Ehrenberg (1838) [37], protists, including photosynthetic flagellates, were placed within the Infusoria, a group assigned to the kingdom Animalia. In contrast, many botanists considered non-motile algae, such as *Closterina* and *Spirogyra*, to be more appropriately classified as plants [38]. To accommodate organisms that could not be clearly assigned to either animals or plants, the kingdom Protoctista [39] or Protista [40] was proposed. These categories were intended to encompass organisms that were intermediate between—or distinct from—animals and plants, and as such, included not only protists but also certain multicellular forms and even bacteria. Separately, Goldfuss (1820) [41] introduced the term Protozoa as a class within the kingdom Animalia, encompassing both protists and simple multicellular animals. Siebold and Stannius (1845) [42] redefined Protozoa to include only unicellular organisms, and with the subsequent inclusion of various groups, the classical classification system exemplified by Bütschli (1880–1889) [43,44] was established (Figure 2). This classical taxonomic framework grouped protists according to morphological and locomotive features, such as Mastigophora (flagellates), Rhizopoda (amoebae), Sporozoa (spore-forming parasites), and *Infusoria* (ciliates). Consequently, the concept of protists of uncertain taxonomic affinity, what we now refer to as PUPAs, scarcely existed at that time. The advent of electron microscopy in the mid-20th century transformed the protist taxonomy by revealing previously unrecognized ultrastructural features [45]. Electron microscopy revealed both the conserved cellular architecture shared by animals, plants, and protists, as well as the remarkable ultrastructural diversity, especially among protists. For example, all eukaryotic flagella basically share a conserved 9 + 2 microtubule arrangement, while protist flagella often have various appendages, such as mastigonemes, scales, or paraxial rods [46]. Significant diversity was also found in the structures of the flagellar transitional region and the flagellar roots. Observations of flagellar appendages and the transition zone suggested a potential phylogenetic affinity among disparate lineages—for instance, tripartite flagellar hairs and spiral fiber in the flagellar transitional region grouped heterokont algae and fungus-like oomycetes, as well as parasitic slopalinids [47,48,49]. Similarly, the presence of discoid mitochondrial cristae indicated a close relationship between kinetoplastids and euglenids [50]. The discovery of cortical alveoli suggested a close affinity between ciliates and dinoflagellates [51,52]. These ultrastructural features laid the foundation of several major taxonomic groups that remain in use today, including Stramenopiles, Euglenozoa, and Alveolata. Ultrastructural studies also revealed charophytes as the closest relatives of land plants, based on similarities in flagellar root systems and cell division [53]. As protist classification gradually shifted from traditional morphology-based systems to those reflecting ultrastructural features [45], it became clear that many previously described protists could not be easily accommodated in the emerging classification schemes [45]. As a result, a large number of unicellular protists, especially heterotrophic flagellates, came to be regarded as of uncertain taxonomic position (i.e., PUPAs) [54].

Subsequent advances in molecular phylogenetic analyses demonstrated that protists are a highly polyphyletic assemblage, distributed across the eToL [55,56,57]. Based on these findings, eukaryotes were divided into “supergroups,” and animals and plants were encompassed within each of them, together with their related unicellular protists [24,25]. One of the most widely used molecular markers for reconstructing eukaryotic phylogeny has been SSU rDNA. This gene is universally present in all eukaryotic organisms and contains both conserved and variable regions that provide phylogenetic signals for resolving relationships among both distantly and closely related lineages. SSU rDNA phylogenies contributed greatly to resolving the phylogenetic positions and taxonomic uncertainties of PUPAs (Table 1). For example, pioneering studies based on morphological data could not clarify the phylogenetic affiliation of *Multicilia*, a multiflagellated protist [54,58,59,60]. Finally, a molecular phylogenetic analysis using SSU rDNA demonstrated that this organism belongs to Amoebozoa [61]. Similarly, *Ebria*, a flagellate with a siliceous skeleton, was variably classified in Dinoflagellata, Opalozoa, or treated as of uncertain affiliation [54,62,63]. A subsequent SSU rDNA phylogenetic analysis revealed that it belongs to Cercozoa [64].

Although SSU rDNA-based phylogenetic analysis has been widely used for inferring the evolutionary relationships among eukaryotes, it has not resolved all their placements in the eToL. Many protists were still treated as PUPAs even after SSU rDNA phylogeny was introduced as one of the standard procedures for eukaryotic classification (Figure 2; see Table 3 in Adl et al. 2019 [26]). The failures in elucidating the taxonomic homes of PUPAs were often due to the absence of sequence data, which in turn reflected either the rarity of these organisms in natural samples [32,80] or the difficulty of culturing them (see Section 3 for examples of such environmentally rare lineages). In other cases, phylogenetic analyses based on a single locus were insufficient to resolve deep evolutionary relationships (e.g., Brugerolle et al. 2002; Klaveness et al. 2005 [81,82]). Resolving the phylogenetic position of such lineages has required further development in analytical tools and sequencing technologies.

## 3. PUPAs Found Their Taxonomic Homes Through Large-Scale Phylogenetic Analyses

Recent advances in sequencing technologies have dramatically enhanced our ability to elucidate the phylogenetic positions of PUPAs [83]. As mentioned in the previous section, certain PUPAs cannot be confidently placed within the eToL using single phylogenetic markers, such as SSU rDNA, alone [80,81,82,84]. In general, the phylogenetic signal in a single marker is limited. Thus, depending on how ancient the phylogenetic split of interest is, single-locus analyses may be insufficient to resolve deep splits with accuracy. One powerful approach to overcome this limitation is “phylogenomics,” analyses of phylogenetic alignments comprising multi-loci data, also often termed as “supermatrices” [85,86,87]. Since the 2000s, improvements in high-performance computing have enabled the analysis of supermatrices. At the same time, the development of massively parallel sequencing (MPS; also known as next-generation sequencing or NGS) technologies, beginning around 2005, provided a practical means to obtain large amounts of sequence data from samples at reasonable cost. This innovation marked a paradigm shift in phylogenetic studies, including those focused on PUPAs. Over the past two decades, both sequencing instruments and related methodologies have undergone continuous updates. Today, it is possible to generate high-quality sequencing libraries from extremely small amounts of DNA or RNA, on the order of picograms, or even from a single cell. The advance in sequence technology is particularly significant for PUPAs, many of which, at least currently, are uncultivable and exist only as rare or precious single-cell samples. The ability to recover massive sequence data from a single cell or a small number of cells has markedly contributed to the recent acceleration in resolving the diversity and evolution of eukaryotes. Below, we highlight key phylogenomic studies that utilized sequence data generated through MPS technologies and successfully solidified the positions of PUPAs and, by extension, the eToL.

### 3.1. Barthelonids as a Deep-Branch Lineage in Metamonada

Among the many PUPAs, *Barthelona* represents a typical case in which the position in the eToL was solidified by the phylogenomic approach. The first report of a *Barthelona* species (*B. vulgaris*) was made by Bernard et al. (2000) [88], who isolated it from marine sediment samples in Australia. However, no molecular data or ultrastructural observations (e.g., electron microscopy) were conducted at the time; the species was described solely through morphological observations. The absence of detailed morphological characteristics or molecular data was understandable, given the study’s focus on showcasing protist diversity in low-oxygen and anoxic environments. Subsequent isolates from Korea and Australia were also described based only on morphology, without molecular data [89,90].

**Figure 3:**
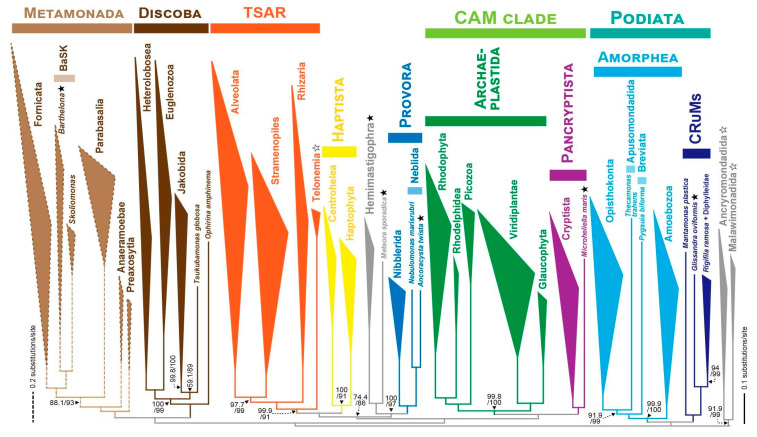
**The maximum likelihood (ML) phylogeny of 162 taxa represents the currently recognized diversity of eukaryotes.** This phylogenetic tree was inferred from the supermatrix analyzed in Yazaki et al. (2025) [32], but after removing 5 redundant species and adding 35 new species, followed by ortholog selection performed using PhyloFisher v1.2.13 [98]. Single-protein alignments were generated with MAFFT v7.520 [99], and alignment trimming was carried out using BMGE v1.12 [100]. The final supermatrix comprised 348 proteins (82,455 amino acid positions in total). The ML tree search, coupled with branch support calculations using UFBoot2 and SH-aLRT (1000 replicates), was conducted using IQ-TREE v2.3.6 [101], under the LG + G + C60 + F model. In the figure, the support values of SH-aLRT and UFBoot2 are shown on the right and left, respectively, divided by a dash. In the case of the bipartitions receiving full support value from both SH-aLRT and UFBoot2, no value was indicated. The PUPAs discussed in Section 3 and Section 4 were marked by closed and open stars, respectively.

Furthermore, the morphological traits of *Barthelona* species did not indicate any close affiliation with known eukaryotic groups [88,89,90], and as such, they remained classified as PUPAs for many years. The “PUPA status” of *Barthelona* was changed with the work conducted by us and colleagues, which reported and analyzed *Barthelona*-like cells isolated from multiple geological locations [91]. The stable laboratory cultures of *Barthelona* species enabled us to generate molecular data since their initial descriptions [88,89,90]. A SSU rDNA-based phylogenetic analysis confirmed that these isolates are monophyletic; however, the precise position of the *Barthelona* species as a whole remained unresolved. A phylogenomic analysis based on a supermatrix of 148 proteins from one of the isolates revealed that this lineage branched with Fornicata, a group of Metamonada constituted exclusively by microaerophilic or anaerobic taxa. This finding has important implications for understanding the reductive evolution of mitochondria and anaerobic ATP metabolism within Fornicata, particularly regarding mitochondrion-related organelles. More recent studies have demonstrated that *Skoliomonas* species isolated from worldwide soda lakes form a robust sister group to *Barthelona* in a phylogenomic analysis of 174 proteins [92,93]. The clades of *Barthelona* and *Skoliomonas* are collectively referred to as “BaSK,” and this grouping was also reconstructed in the phylogenomic analyses of 340 proteins (Figure 3).

### 3.2. *Glissandra* Expanded Our Knowledge of the Diversity of CRuMs

Another example of PUPAs that has been known only from morphological data for a long time is *Glissandra*. The first two species, *G. similis* and *G. innuerend*, were isolated from marine sediments in Australia and described solely based on morphological observations [90,94]. In 2013, a *Glissandra*-like organism, later named *G. oviformis*, was rediscovered on the surface of algae in a marine lake in the Republic of Palau and was successfully established as a laboratory culture for both microscopic and molecular work [32]. The SSU rDNA analysis failed to pinpoint its phylogenetic positions, but the transcriptome data of *G. oviformis* permitted a 348-protein phylogenomic analysis, which placed *G. oviformis* within CRuMs (see also Figure 3). CRuMs is a “supergroup-compatible” clade, first proposed in 2018 based solely on a phylogenomic analysis [95], and no clear morphological synapomorphy has been proposed. Considering the ultrastructural information from *G. oviformis*, together with that from other members of CRuMs [81,96,97], the pellicle underlying the plasma membrane and an internal sleeve at the flagellar base were first proposed as the potential ancestral traits inherited from the common ancestor of this clade [32].

### 3.3. Emergence of a New Supergroup Encompassing Hemimastigophorans, *Meteora*, and Provora

In the previous sections, we introduced the PUPAs that represent previously overlooked branches associated with the previously recognized clades in the eToL. On the other hand, some PUPAs have even led to the discovery of entirely novel, high-level clades, such as supergroups. A prominent example is Hemimastigophora, a group of multi-flagellated protists first described in 1988 [102]. In 1993, ultrastructural observations revealed similarities in pellicle and nuclear morphology to euglenozoans, yet no clear phylogenetic affiliation could be determined at the time [103]. It took more than 20 years for Hemimastigophora to be rediscovered and investigated in the molecular framework [30]. The SSU rDNA analysis of two hemimastigophorans—*Hemimastix kukwesjijk* and *Spironema* cf. *multiciliatum*—showed no clear affinity to any known eukaryotic lineage. Likewise, the phylogenetic position of hemimastogphorans could not be settled by the single-cell transcriptome-based phylogenomic analyses of 351 proteins. Thus, Lax et al. (2018) [30] proposed Hemimastigophora as the representative of a completely novel and independent eukaryotic lineage.

Unlike other PUPAs known before molecular analyses gained popularity, *Ancoracysta twista* was an example of the PUPA in a phylogenomic era. Janouškovec et al. (2017) [29] reported this protist, which exhibits unique morphological characteristics, and could not confidently place it in the eToL, even after analyzing a supermatrix of 201 proteins. More recently, *A. twista* appeared to be the tip of the iceberg in a group of protists previously unknown to science, and the clade containing *A. twista* as a whole was designated as Provora [31]. Tikhonenkov et al. (2022) [31] proposed Provora as an eukaryotic “supergroup,” but their phylogenomic analysis based on 320 proteins left the placement of Provora relative to other major assemblages uncertain.

In a separate line of work from *A. twista* and Provora (see above), a recent analysis clarified that *Meteora sporadica*, a long-standing PUPA, represents a sister lineage of Hemimastigophora [35]. *M. sporadica*, which moves using arm-like structures protruding from both sides of the cell, was initially isolated from Mediterranean Sea sediments and described in 2002 [104]. The SSU rDNA phylogeny failed to clarify the position of *M. sporadica* in the eToL [80]. Eventually, some of us succeeded in re-isolating and establishing multiple laboratory cultures of *M. sporadica*, enabling us to compose and analyze a supermatrix of 254 proteins [35]. In the resultant phylogenetic tree, *M. sporadica* formed a clade with Hemimastigophora with high statistical support. *M. sporadica* and Hemimastigophora were then connected to *A. twista*, yet the statistical support for the particular bipartition was inconclusive. Intriguingly, more recent phylogenomic analyses, particularly those incorporating *M. sporadica* and its relative, suggested an even larger “supergroup” comprising (at least) Hemimastigophora, *M. sporadica* (with and without its relative), and Provora, with a specific affinity between the former two [28,33,35]. Indeed, the phylogenomic analysis of 348 proteins also yielded the clade comprising Hemimastigophora, *M. sporadica*, and Provora (Figure 3).

### 3.4. *Microheliella* Acts as a “Phylogenetic Matchmaker” Between Archaeplastida and Cryptista

While the discovery of entirely novel eukaryotic lineages—as exemplified by *Meteora*, Hemimastigophora, and Provora—has been critical for expanding the eToL, PUPAs that serve to bridge major established lineages are equally significant. A representative case is *Microheliella*. *Microheliella maris*, which was isolated from the marine sediment from the Ebro Delta in Spain, was first reported as a heliozoan-like protist with radiating axopodia [105]. This initial report included morphological observations under light microscopy and SSU rDNA phylogenetic analysis, but the phylogenetic affiliation of the organism was unresolved. Yabuki et al. (2012) [84] conducted detailed electron microscopy and demonstrated that the cellular structure of *M. maris*, which was the laboratory culture strain, differed from those of known heliozoans. The same study conducted a phylogenetic analysis of an alignment comprising SSU rDNA, large subunit rDNA, and heat-shock protein 90 that hinted at a possible relationship with *P. bilix* [84]. Later, a larger-scale, transcriptome-based phylogenomic analysis considering 187 proteins was performed, but could not solidify the position of *M. maris* in the eToL [106]. Finally, a study involving some of us generated new transcriptome data for *M. maris* that allowed us to generate and analyze an even larger supermatrix of 319 proteins [107]. In this analysis, *M. maris* was grouped with Cryptista (including *P. bilix* [108]), and the clade comprising *Microheliella* and Cryptista was designated as Pancryptista.

The incorporation of *Microheliella* into phylogeomic analyses helped to resolve a deeper split in the eToL. The putative false affinity between cryptophytes and Rhodophyta often hindered the recovery of the monophyly of Archaeplastida in phylogenomic analyses (e.g., Burki et al. 2016; Janouškovec et al. 2017; Gawryluk et al. 2019 [29,109,110]). Fortunately, non-photosynthetic lineages, which position basal to cryptophytes (i.e., *Microheliella* and *P. bilix*) and Rhodophyta (i.e., Rhodelphida and Picozoa), were found to suppress the false affinity between cryptophytes and Rhodophyta, resulting in the recovery of the sister relationship between Archaeplastida and Pancryptista (*Microheliella* plus Cryptista) (proposed as CAM clade; Yazaki et al. 2022 [107]). The series of studies described above underscores the potential role that PUPAs play in filling phylogenetic gaps and bridging two (or more) major assemblages in the eToL.

**Figure 4:**
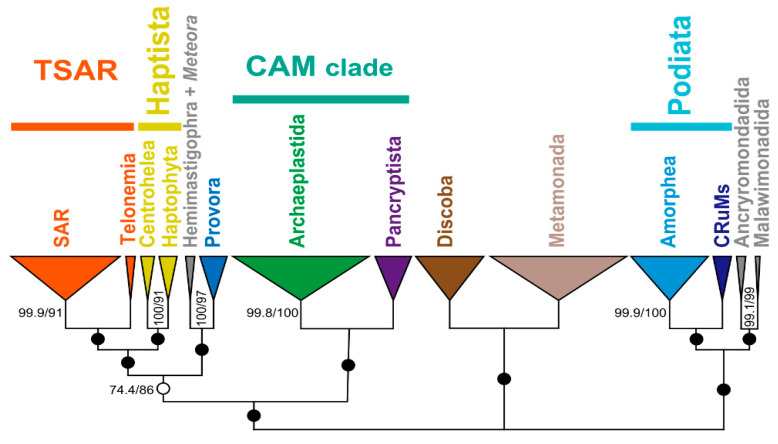
**Assessments of selected backbone bipartitions in the eToL by an approximately unbiased test.** This cladogram was created by modifying the maximum likelihood tree inferred from the supermatrix comprising 348 proteins shown in Figure 3. The triangles represent major clades recovered from the ML analysis. The widths of triangles were scaled to the number of OTUs included in the clades. The support values (SH-aLRT/UFBoot2) are shown only for the bipartitions that did not receive full support from either method. For each of the 11 bipartitions selected, the ML and two alternative trees generated by nearest-neighbor-interchange on the particular bipartition were subjected to an approximately unbiased (AU) test. A series of AU tests was automated by AUTOEB v1.1.1 [111]. The settings of AUTOEB were set as the default. In the case of both alternative trees being rejected, we regarded the bipartition of interest as “resolved,” and marked it by a closed circle. Only 1 of the 11 bipartitions examined was judged as “unresolved,” highlighted by an open circle.

## 4. Uncertainties in the eToL Related to PUPAs

There are a few PUPAs that have been examined in the phylogenomic framework, but their phylogenetic home currently remains unsettled. One of those PUPAs, Telonemia, has been regarded as the closest relative of SAR. This group has been well characterized ultrastructurally [112,113], and its morphology has been linked to those of Alveolata, Stramenopiles, and Rhizaria, in concordance with the phylogenetic affinity to SAR recovered in phylogenomic analyses [31,32,33,114,115,116]. This relationship was also recovered in the phylogenomics analysis conducted in this review (Figure 3). In addition, the union of Telonemia and SAR was regarded as “resolved” by AUTOEB [111], which examined the ML and two alternative tree topologies generated by nearest-neighbor-interchange on the bipartitions of interest, and rejected both of the alternative trees by an approximately unbiased test [117] (labeled by closed circles in Figure 4). Yet, what we should pay attention to is that the union of Telonemia and SAR was not always recovered in past phylogenomic analyses [35,98,107], leaving some room for the position of Telonemia in the eToL. Notably, in one of the most recent phylogenomic analyses, Telonemia formed a clade with Haptophyta and Centrohelea, exhibiting a specific affinity to the former [28]. The authors did not explicitly explore the reason why Telonemida interrupted the Haptista clade instead of grouping with SAR. If the results presented in Zlatogursky et al. (2025) [28] were genuine (i.e., the affinity between Telonemia and SAR recovered in the past analyses was false), the increment of a novel, deep-branching telonemid species triggered the change in the position of Telonemia in the eToL. The above notion was deduced from a comparison between the two most recent phylogenomic trees of eukaryotes, one presented in Zlatogursky et al. (2025) [28] and the other in Čepička et al. (2025) [33], in which overall taxon samplings were similar to each other, except for Telonemia; the former and latter tied Telonemia to Haptophyta and SAR, respectively. We need to revisit the position of Telonemia in the eToL in future phylogenomic analyses with an improved sampling in Telonemia, Haptophyta, and Centrohelea, particularly novel members branching at the base of those clades. It may also be necessary to re-interpret the morphological characteristics of Telonemia depending on the position of this lineage.

A substantial amount of morphological data has been accumulated for Ancyromonadida and Malawimonadida so far. The members of Malawimonadida are equipped with flagella associated with vanes and complex flagellar apparatuses that support feeding grooves on the ventral side of the cell [118,119,120]. Those features were regarded as compelling evidence for Malawimonadida as typical excavates [121]. The morphological data of Ancyromonadida are less determinate, but some features hint at the connection to excavates [122,123,124]. Nevertheless, either of the two PUPAs showed no clear affinity to any lineages, regardless of whether they contained typical excavates, in recent phylogenomic analyses [27,28,31,32,33,35,98,107]. Rather, as shown in Figure 3, unrooted phylogenomic analyses often split Ancyromonadida, Malawimonadida, Amorphea, and CRuMs on one hand, and the rest of eukaryotes on the other in the eToL. Significantly, the dichotomy in the unrooted eToL was judged as “resolved” by AUTOEB (labeled as closed circles in Figure 4). In those trees, Ancyromonadida and Malawimonadida were either paraphyletic or formed a clade at the base of Amorphea and CRuMs (i.e., Podiata [125]). To our knowledge, no study, except for two recent ones [27,114], has examined explicitly [27,115] the relationship among Ancyromonadida, Malawimonadida, and Podiata. In an AU test conducted in Harada et al. (2024) [116], among the nine test tree topologies subjected to an AU test, only two trees, one (ML tree) bearing Podiata connected with the clade comprising Ancyromonadida and Malawimonadida, and the other in which Malawimonadida and then Ancyromonadida were sequentially connected to Podiata, failed to be rejected at the 5% level. Torruella et al. (2025) [27] also recovered the ML tree with the clade comprising Ancyromonadida and Malawimonadida, which, as a whole, was sister to Podiata; however, a certain possibility for a direct connection between Malawimonadida and Podiata, excluding Ancyromonadida, was seemingly left open. So far, previously published phylogenomic studies have suggested a possible affinity among Ancyromonadida, Malawimonadida, and Podiata, although their precise phylogenetic relationship requires confirmation. As seen in a recent study on Telonemia (see above), the positions of Ancyromonadida and Malawimonadida may be revised drastically when novel protists, which are closely related to but clearly distinguishable from the known ancyromonads and malawimonads, are included in future phylogenomic analyses.

We expect that some of the current PUPAs will hold critical clues for resolving the eToL. As seen for CRuMs [32,95] and the clade comprising *Meteora*, Hemimastigophora, and Provora [30,31,35], they may represent novel groups with no clear affinity to any of the currently recognized major lineages in the eToL. However, PUPAs do not necessarily have to be entirely novel. We can achieve a better-resolved eToL by incorporating the PUPAs and novel protists, which are basal to the established lineages, into phylogenomic analyses, as seen for *M. maris* and Rhodelphida, which helped unite Cryptista and Archaeplastida [107]. Furthermore, Rhodelphida, as well as Picozoa, which are non-photosynthetic relatives of Rhodophyta, were also found to be significant in inferring the internal branching pattern in Archaeplastida by suppressing a phylogenetic artifact stemming from fast-evolving positions in a supermatrix [126].

## 5. Beyond PUPAs: Novel Protists for Elucidating the eToL

Future studies on PUPAs are significant to elucidate the eToL, but they may not sufficiently represent the as-yet-unveiled diversity of eukaryotes. There is most likely a large number of novel protists that have been entirely unknown to science in natural environments. Environmental DNA (eDNA) analyses have been regarded as one of the powerful tools for evaluating the diversity of organisms that thrive in natural samples of interest. In our recent work [32], we found that the SSU rDNA amplicon sequence of *G. oviformis* had been misannotated as that of the red alga *Porphyra capensis* in the EukBank database. Thus, it is difficult to conclude the organismal origins of the amplicon sequence variants (ASVs) bearing nucleotide identities of less than 75% to the SSU rDNA in the GenBank databases, considering the basis of the (mis)annotation of the *G. oviformis* amplicons to *P. capensis* in the Eukbank database [32]. Surprisingly (or not), even if the minimum nucleotide sequence identity for annotation is set more stringently—i.e., equal to or greater than 85%—the organismal origins of almost 40% of ASVs in the Eukbank database, namely 133,313 ASVs, failed to meet the criterion for annotation. A closer examination of the EukBank dataset (see Table 2) reveals that a substantial proportion of ASVs detected in marine environments lack close matches to GenBank entries. When ASVs with less than 85.0% similarity are considered, nearly 40% of marine ASVs show low affinity to known sequences. Likewise, 10–30% of ASVs in inland environments appeared to bear less than 85.0% similarity to known sequences. These results imply that both marine and land environments harbor a larger proportion of previously uncharacterized protists (potentially including PUPAs). Thus, land soils could be a promising source of novel lineages, including those similar to a previous PUPA lineage, hemimastigophorans, constantly discovered in soil samples [30,102,103].

It is unquestionable that the metagenomics approach is powerful in characterizing diverse prokaryotes, thereby bypassing the steps of laboratory culturing. Nevertheless, this approach may or may not be efficient for hunting PUPAs or novel protists (PUPAs/NPs). Compared to prokaryotic genomes, genes in eukaryotic (nuclear) genomes are often interrupted by a (potentially large) number of introns and are more sparsely distributed. It is also likely that various repetitive sequence elements occupy a large proportion of eukaryotic genomes. Thus, unless extremely compact genomes, the information in metagenome-assembled genomes (MAGs) obtained by currently available technologies may not be sufficient to judge with confidence which MAGs originated from PUPAs/NPs. Future advances in the technologies related to metagenomics may achieve efficient amplification of much longer MAGs from a much smaller amount of eDNA samples, enabling us to help hunt PUPAs/NPs by this particular approach. We emphasize here that, even after the metagenomic approach becomes applicable to study PUPAs/NPs, efforts to culture them in laboratories, which unify sequence data and cellular identities, including ultrastructural information, should be continued.

To address the issues in the eToL (see above), it is crucial to include the PUPAs/NPs that split “long naked” branches, which lead to the clades of Telonemia, Centrohelea, Haptophyta, Ancryomonadida, and Malawimonadida in phylogenomic analyses. The particular PUPAs/NPs we need here are those for which phylogenetic affiliations cannot be pinpointed by single-locus (e.g., SSU rDNA) analyses. In line with this discussion, a potentially novel group of algae, recently unveiled through metagenome analyses, is a good candidate for the previously overlooked relatives of Haptophyta [34]. Once the PUPAs/NPs, which are closely related to but clearly distinct from the five lineages mentioned above, are included in future phylogenomic analyses, we can more precisely retrace how the current eukaryotic diversity has been shaped.

## 6. Conclusions

As discussed in this review, the positions of PUPAs (and novel protists) are critical in resolving the eToL. First of all, they are anticipated to fill the “holes” in the currently recognized diversity of eukaryotes. In addition, high-quality sequence data generated from the laboratory cultures of PUPAs/NPs are significant for rigorous phylogenomic reconstruction of the eToL. In general, bipartitions in trees inferred from supermatrices receive high or often full support values, such as non-parametric bootstrap support values and Bayesian posterior probabilities. Nevertheless, some of the highly supported bipartitions reconstructed may or may not be derived from methodological artifacts in tree reconstruction. By including additional PUPAs/NPs in future analyses, we will infer the eToL more accurately by splitting long branches or allowing them to surrogate problematic taxa that bias phylogenetic inferences.

Finally, it is indispensable to pinpoint the root position for future challenges in inferring a more accurate architecture of the eToL. Published studies on PUPAs/NPs successfully expanded previously recognized groups or coalesced two of them. Our efforts studying PUPAs/NPs can narrow down the possible positions of the root, as it is unlikely to lie within a currently recognized or a yet-to-be-defined supergroup. In the case of a PUPA/NP uniting two supergroups across the putative root, it would be a good reason to discard the particular hypothesis for the eukaryotic root.

## Figures and Tables

**Table 1:** Groups of protists for which taxonomic affiliations have been updated by molecular phylogenetic analyses using SSU rDNA since 2005.

Name	Taxa Assumed by Morphological Data	Affiliations Identified by SSU rDNA Phylogeny
*Cyranomonas*	Unclassified	Novel clade CU (Cercozoa) [65]
Ebriida	Dinoflagellata Silicoflagellata Unclassified	Thecofilosea (Cercozoa) [64]
*Meringosphaera*	Chrysophyceae (Stramenopiles) Centroplasthelida Unclassified	Environmental marine clade NC5 (Centroplasthelida) [66]
*Multicilia*	Amoebozoa Rhizomastigina Unclassified	Variosea (Amoebozoa) [61,67]
Parmales	Chrysophyceae (stramenopiles)	Bolidophyceae (Stramenopiles) [68]
*Pseudophyllomitus*	Unclassified	MAST-6 (Stramenopiles) [17]
*Quadricilia*	Unclassified	Novel clade 2 (Cercozoa) [69]
*Solenicola*	Xanthophyceae (Stramenopiles) Unclassified	MAST-3 (Stramenopiles) [70]
*Metopion*	Unclassified	Metopiida (Cercozoa) [71]
*Pleurostomum*	Unclassified	Heterolobosea (Discoba) [72]
Vampyrellidae	Sarcodina	Novel clade 8 (Endomyxa) [73]
*Rhizomastix*	Mastigophora	Archamoebae (Amoebozoa) [74]
*Protaspa*	Dinoflagella Cercozoa Euglenozoa	Thecofilosea (Cercozoa) [64]
*Artodiscus*	Amoebozoa Cercozoa Sarcodina	Variosea (Amoebozoa) [67]
*Clautriavia*	Unclassified	Imbricatea (Cercozoa) [75]
*Abollifer*	Unclassified	Imbricatea (Cercozoa) [76]
Amphitremida	Sarcodina Thecofilosea (Cercozoa)	Labyrinthulomycetes (Stramenopiles) [77]
*Paramonas*	Unclassified	Bicosoecida (Stramenopiles) [78]
*Reticulamoeba*	Granofilosea (Cercozoa) Proteomyxidea Unclassified	Granofilosea (Cercozoa) [79]

**Table 2:** Summary of ASVs with low similarity across environments in the EukBank 18S V4 dataset.

Environment	Percentage of ASVs with Similarity < 85.0%
Marine water	16.73%
Marine sediment	19.24%
Marine organism	13.16%
Marine ice	5.70%
Land water	8.50%
Land soil	29.84%
Land sediment	22.00%
Land organism	11.04%
Land freshwater	17.36%

## Data Availability

The original contributions presented in this study are included in the article/Supplementary Material. Further inquiries can be directed to the corresponding author.

## Supplementary Materials

The following supporting information can be downloaded at: https://www.mdpi.com/article/10.3390/microorganisms13081926/s1, Supplementary Data S1. Figure S1 (*162otuTree_descriptive.pdf*): The detailed maximum likelihood (ML) phylogeny of 162 taxa without clades being collapsed (corresponding to Figure 3). Supplementary Data S2. The concatenated amino acid alignment of 348 proteins from 162 taxa (*162otu348genes.fas*) used for the ML phylogenetic analyses. Supplementary Data S3. The maximum likelihood (ML) tree (*result.tree*) inferred from the 348-protein alignment in Supplementary Data S2.
