# Supplementary figures and images for: Protists with Uncertain Phylogenetic Affiliations for Resolving the Deep Tree of Eukaryotes
Source: Microorganisms. 2025 Aug 18;13(8):1926. doi: 10.3390/microorganisms13081926 (PMC12388492; doi:10.3390/microorganisms13081926)

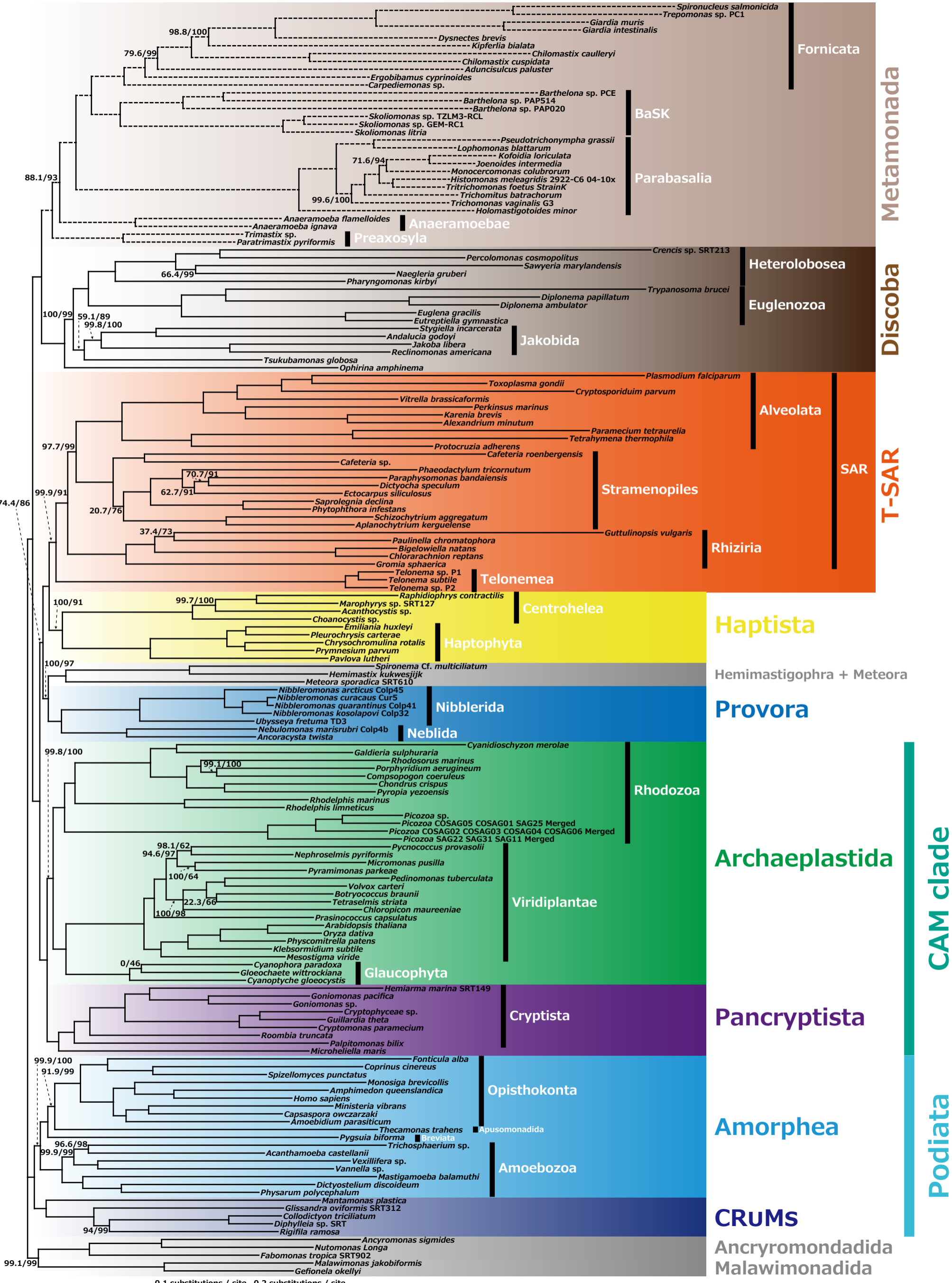

Supplement: Supplementary file 1 [file microorganisms-13-01926-s001.zip › Supplementary Data S1-162otuTree_descriptive.pdf]
